# Antimicrobial and Antiproliferative Potential of *Anadenanthera colubrina* (Vell.) Brenan

**DOI:** 10.1155/2014/802696

**Published:** 2014-06-30

**Authors:** Rennaly de Freitas Lima, Érika Ponchet Alves, Pedro Luiz Rosalen, Ana Lúcia Tasca Gois Ruiz, Marta Cristina Teixeira Duarte, Vivian Fernandes Furletti Góes, Ana Cláudia Dantas de Medeiros, Jozinete Vieira Pereira, Gustavo Pina Godoy, Edja Maria Melo de Brito Costa

**Affiliations:** ^1^Department of Dentistry, Paraíba State University (UEPB), 58429-500 Campina Grande, PB, Brazil; ^2^Department of Pharmacology and Anesthesiology, Piracicaba School of Dentistry, University of Campinas (UNICAMP), 13414-903 Piracicaba, SP, Brazil; ^3^Center for Chemical, Biological and Agricultural Research, University of Campinas (UNICAMP), P.O. Box 6171, 13083-970 Campinas, SP, Brazil

## Abstract

The aim of the present study was to perform an *in vitro* analysis of the antimicrobial and antiproliferative potential of an extract from *Anadenanthera colubrina* (Vell.) Brenan (angico) and chemically characterize the crude extract. Antimicrobial action was evaluated based on the minimum inhibitory concentration (MIC), minimum bactericidal/fungicidal concentration, and the inhibition of formation to oral biofilm. Cell morphology was determined through scanning electron microscopy (SEM). Six strains of tumor cells were used for the determination of antiproliferative potential. The extract demonstrated strong antifungal activity against *Candida albicans* ATCC 18804 (MIC = 0.031 mg/mL), with similar activity found regarding the ethyl acetate fraction. The extract and active fraction also demonstrated the capacity to inhibit the formation of *Candida albicans* to oral biofilm after 48 hours, with median values equal to or greater than the control group, but the difference did not achieve statistical significance (*P* > 0.05). SEM revealed alterations in the cell morphology of the yeast. Regarding antiproliferative activity, the extract demonstrated cytostatic potential in all strains tested. The present findings suggest strong antifungal potential for *Anadenanthera colubrina* (Vell.) Brenan as well as a tendency toward diminishing the growth of human tumor cells.

## 1. Introduction

The high prevalence rates of adverse oral conditions and the limitations of available treatment methods have led to increasing interest among the scientific community in alternative therapies [1]. This interest has fueled research into natural compounds with adequate effectiveness and fewer adverse effects in the control of oral biofilm, which is responsible for the development of conditions such as tooth decay and candidiasis [2].

Bacterial and fungal infections pose a challenge to researchers and clinicians, especially in immunocompromised patients. This situation is further complicated by the development of resistance to conventional medications on the part of a large number of microorganisms. Therefore, studies are needed for the discovery of safe, stable drugs from sources found in nature that are effective against resistant bacteria and fungi [3]. Such drugs could also benefit a greater number of individuals due to the low cost and greater availability [4].

Medicinal plants have become an important source for the discovery of novel antimicrobial agents. Raw extracts from plants and isolated substances in these extracts have demonstrated activity against a number of pathogens [5]. It is also important to determine the potential of natural products with biological activity regarding possible harm to normal cells as well as damage to tumor cells to establish possible therapeutic uses [6].

Multidisciplinary studies, especially those uniting phytochemistry and pharmacology, have become increasingly necessary due to the use of medicinal plants throughout the world [7].* Anadenanthera colubrina* (Vell.) Brenan, commonly known as “angico”, is a tree belonging to the subfamily Mimosoideae (Leguminosae) that occurs in different biomes in Brazil, Bolivia, Argentina, Paraguay, and Peru [8, 9].* A. colubrina *is reported to be one of the most widely used plants in folk medicine and is employed to treat respiratory problems, inflammation, diarrhea, cough, bronchitis, influenza, and toothache [10–14]. The bark is the most commonly used part of the plant, the availability of which is not limited by variations in climatic conditions [15].

The aim of the present study was to perform an* in vitro *analysis of the antimicrobial and antiproliferative potential of the crude extract and fractionated extracts from* Anadenanthera colubrina* (Vell.) Brenan (angico).

## 2. Materials and Methods

### 2.1. Preparation of Hydroalcoholic Extract and Fractionation

*A. colubrina *was collected in the month of September from the Bodocongó Hills in the city of Queimadas, which is located in the Borborema tableland of the eastern Cariri microregion of the semiarid region of the state of Paraíba, Brazil (7° 22′ 25′′S, 35° 59′ 32′′W). A voucher specimen was deposited in the Manuel de Arruda Câmara Herbarium of the Paraíba State University (Campus I, Campina Grande, Paraíba, Brazil) under* n*° 667/ACAM.

The bark (100 g) was dried, ground, and immersed in 80% ethanol (250 mL) at room temperature for 48 hours. The resulting mixture was filtered and the residues were immersed in 80% alcohol two additional times. The three final liquid phases were concentrated in a rotary evaporator and freeze-dried. Liquid-liquid partition was used to fractionate the extract based on the polarity gradient. Hexane, chloroform, ethyl acetate, and aqueous fractions were obtained and submitted to thin-layer chromatography using anisaldehyde as the reagent. The fractions were then incubated at 100°C for five minutes.

### 2.2. Microorganisms and Susceptibility Test

The following microorganisms were used:* Streptococcus mutans *ATCC 25175*, Streptococcus sanguinis *ATCC 10557*, Pseudomonas aeruginosa *ATCC 27853,* Enterococcus faecalis* ATCC 29212, and* Candida albicans *ATCC 18804. Antimicrobial activity of the hydroalcoholic extract and fractionated extracts of* Anadenanthera colubrina* (Vell.) Brenan was identified, by microdilution in broth, determining the minimum inhibitory concentration (MIC), minimum bactericidal concentration (MBC), and minimum fungicidal concentration (MFC) following the norms established by the Clinical and Laboratory Standards Institute [16, 17].

The test was carried out in 96-well microplates containing 100 *μ*L/well of the specific culture medium (brain heart infusion (BHI; Difco, Franklin Lakes, NJ, USA) for bacteria and RPMI 1640 (Angus Buffers & Biochemicals, Niagara Falls, NY, USA) for yeast). The substances were diluted in 40% alcohol (8 mg/mL) and transferred to the first well and serial dilutions were then performed to obtain concentrations between 15.62 and 2000 *μ*g/mL. Chlorhexidine 0.12% (Sigma-Aldrich) and nystatin (Sigma-Aldrich) were used as the positive controls and 40% alcohol was used as the negative control. 40% alcohol was tested and showed no antimicrobial activity against the microorganism studied. The bacterial inocula (1.0 × 10^6^ colony-forming units (CFU)/mL) and fungal inocula (5.0 × 10^3^ CFU/mL) were added to the wells and the plates were incubated at 37°C for 24 hours. The MIC was defined as the lowest concentration of the extract or fraction that inhibited visible microbial growth, which was confirmed with 0.01% resazurin (Sigma-Aldrich, St. Louis, MO, USA) for bacteria and by the change in color of the RPMI 1640 for yeast.

For the determination of MBC/MFC, an aliquot (50 *μ*L) from each well with concentrations greater than the MIC was subcultured on BHI agar for bacterial and Sabouraud dextrose agar for yeast and incubated at 37°C for 24 hours. MBC and MFC were defined as the lowest concentration that inhibited visible growth on the solid medium.

All assays were performed in triplicate in three independent experiments. At the end of the tests, the active fraction of the* A. colubrina *extract was determined.

### 2.3. Analysis of Formation to Biofilm

Biofilms were produced in untreated 96-well plates. For the evaluation of the formation of microbial cells to the biofilm, 100 *μ*L of a microbial suspension containing 10^5^ CFU/mL (yeast) cultivated in RPMI with the addition of 2% sucrose was transferred to the different wells containing concentrations of 15.62 to 2000 *μ*g/mL of the hydroalcoholic extract and active fraction. The plates were incubated at 37°C for 48 hours under agitation (75 rpm). As the negative control, three wells in each plate were submitted to the same procedures without the addition of the treatments. Nystatin was used as the positive control. All assays were carried out in triplicate in three independent experiments.

For the quantification of adhered cells, the wells were rinsed with distilled water and allowed to dry at room temperature for 45 minutes. An aqueous solution of 1% crystal violet (200 *μ*L) was added to each well and left to stand for 45 minutes. The wells were rinsed again with distilled water and destained with 200 *μ*L of 95% ethanol. After 45 minutes, 150 *μ*L of the destained solution from each well was transferred to a new 96-well plate and the amount of crystal violet was measured in a microplate reader (SpectraMax 340 Tunable Microplate Reader; Molecular Devices Ltd.) at 525 nm. Absorbance values of the substances were subtracted from that of the negative control to determine the number of adhered microbial cells [18, 19].

### 2.4. Analysis of Cell Morphology

Biofilms were formed on slides (Lab-Tek, Nunc, Naperville, IL, USA) using the method described above and treated with the extract and active fraction at concentrations capable of inhibiting the microbial growth (MIC/MFC). The cells were fixed in 3% glutaraldehyde in a phosphate buffer solution at room temperature for 12 hours. The biofilm was then dehydrated in increasing concentrations of ethanol (50, 70, 90, and 100%), gold sputtered, and examined using scanning electron microscopy (SEM; JEOL JSM 5600LV, JEOL Tokyo, Japan).

### 2.5. Antiproliferative Test

The proliferative study involved human keratinocytes (HaCaT) and six human tumor lines: NCI-ADR/RES (ovary with phenotype resistance to multiple drugs), 786-O (kidney), NCI-H460 (lung), OVCAR-3 (ovary), HT-29 (colon), and K562 (bone marrow). Cultures were performed in 5 mL of RPMI 1640 medium (Gibco-BRL, Grand Island, NY, USA) supplemented with 10% bovine fetal serum (Gibco-BRL, Grand Island, NY, USA), with the addition of a blend of penicillin and streptomycin (1000 U/mL: 1000 mg/mL; 1 mL/L RPMI). The cultures were placed in 96-well plates (100 *μ*L/well) and exposed to the hydroalcoholic extract and active fraction, diluted in sodium dimethylsulfoxide (DMSO, Sigma-Aldrich, St. Louis, MO, USA) at a concentration of 0.1 g/mL, and incubated at 37°C in a humid atmosphere with 5% CO_2_ for 24 hours. The final concentration of DMSO did not affect cell viability.

Before (plate T_0_) and after (plate T_1_) the addition of the sample, the cells were fixed with 50% trichloroacetic acid and cell proliferation was determined by the quantification of proteins in a spectrophotometer at 540 nm, using sulforhodamine B. Based on the concentration-response curve for each cell line, the GI_50_ (concentration of raw hydroalcoholic extract needed to inhibit growth by 50%) was determined through a linear regression analysis using the Origin 8.0 program (OriginLab Corporation).

### 2.6. Determination on Total Flavonoids, Total Polyphenols, and Condensed Tannins

For the determination of total flavonoid content, a 2% aluminum chloride (AlCl_3_) solution was added to the extract. After 10 minutes, the reading was performed in a spectrophotometer (Shimadzu UV mini-1240) at 415 nm. The calibration curve was determined based on solutions of quercetin. The concentration of flavonoids was expressed in milligram equivalents of quercetin [20].

For the determination of total polyphenol content, the Folin-Ciocalteu 1N reagent was added to an aqueous solution of the extract and the mixture was allowed to stand for two minutes. An aqueous solution of 20% sodium carbonate (Na_2_CO_3_) was then added. After 10 minutes, absorbance was read at 757 nm. The calibration curve was determined based on solutions of gallic acid. The concentration of polyphenols was expressed in milligram equivalents of gallic acid [21].

For the determination of the content of condensed tannins, a vanillin solution was added to the extract, followed by the addition of 37% hydrochloric acid (HCl). The reaction occurred in test tubes placed in a water bath at a temperature of approximately 20°C. The reading was performed at 500 nm. The calibration curve was determined based on solutions of catechin. The concentration of condensed tannins was expressed in milligram equivalents of catechin [22].

All experiments described above were carried out in triplicate.

### 2.7. Statistical Analysis

Statistical analysis was performed with the data from the test on the inhibition of formation to oral biofilm (discrete quantitative variable). The Kolmogorov-Smirnov test revealed nonnormal distribution. Thus, the comparison of substances was performed with the nonparametric Kruskal-Wallis test, with the level of significance set to 5% (*P* < 0.05).

## 3. Results

Table 1 displays the MIC and MBC/MFC for the hydroalcoholic extract of* A. colubrina*. The best activity was found against* Candida albicans.* Based on this finding, the different fractions of the extract were only tested on this species (Table 2). The ethyl acetate fraction achieved the best result (MIC = 31.25 *μ*g/mL), which demonstrated fungistatic action similar to that of the crude extract.

**Figure 1:**
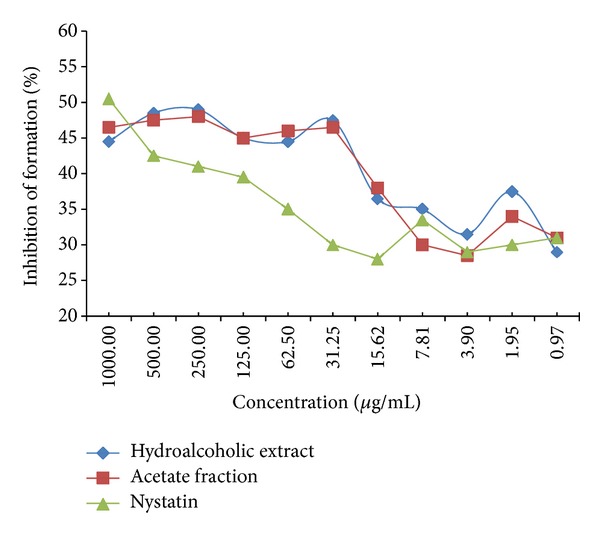
Effect of* Anadenanthera colubrina* Brenan extract, active fraction, and nystatin on mature biofilm (48 h).

The test for the inhibition of the formation to oral biofilm by the* A. colubrina *extract and its ethyl acetate fraction demonstrated equivalent results, with a fair performance until reaching the MIC (31.25 *μ*g/mL). The median values of the crude extract and active fraction were higher than those achieved with nystatin, but the differences did not achieve statistical significance (*P* > 0.05) (Figure 1).

**Figure 2:**
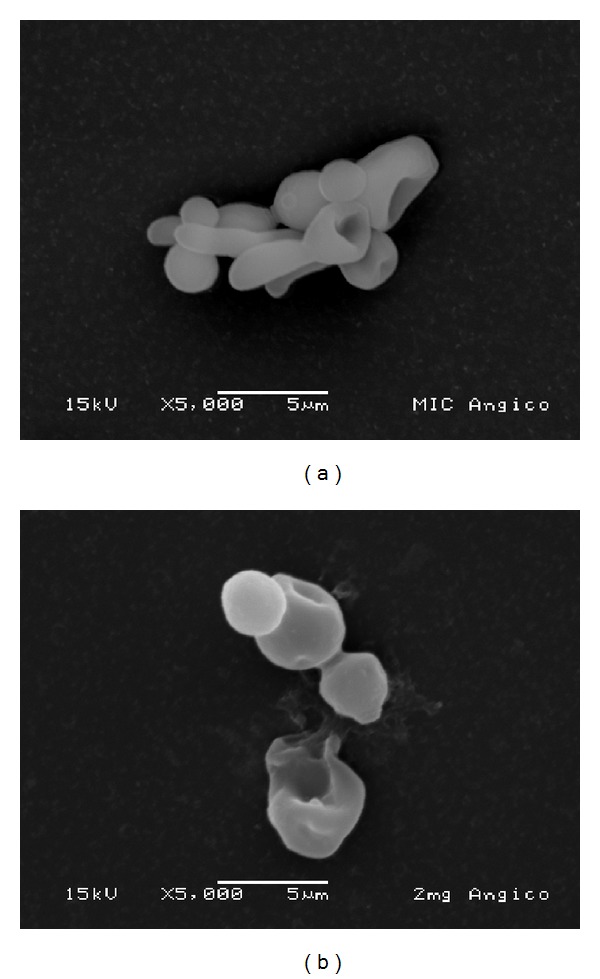
Microphotograph (SEM) of* Candida albicans* biofilm treated with* Anadenanthera colubrina* (Vell.) Brenan extract at concentrations of 31.25 *μ*g/mL (a) and 2000 *μ*g/mL (b) (magnification: 5000x).

**Figure 3:**
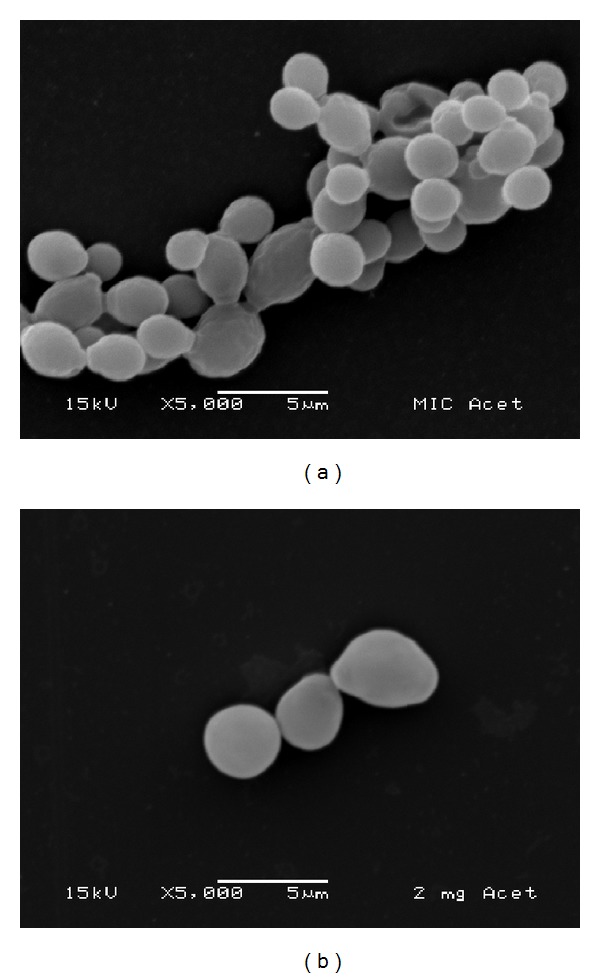
Microphotograph (SEM) of* Candida albicans* biofilm treated with ethyl acetate fraction at concentrations of 31.25 *μ*g/mL (a) and 2000 *μ*g/mL (b) (magnification: 5000x).

**Figure 4:**
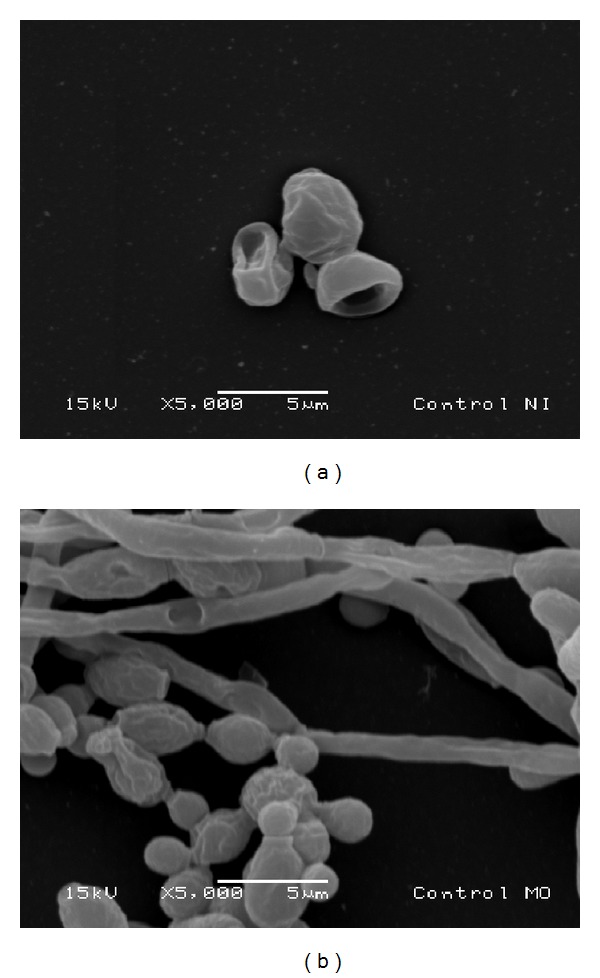
Microphotograph (SEM) of* Candida albicans* biofilm treated with nystatin (a) and negative control (b) (magnification: 5000x).

The SEM images demonstrate the effect of the extract (Figure 2) and its active fraction (Figure 3) at the respective MIC and MFC on* Candida albicans *biofilm. These figures suggest the occurrence of alteration in cell wall of* Candida albicans*, exhibited the occurrence of de-structuring of the cell morphology with the use of the test substances, and predominance of the ovoid blastospores form and few pseudohyphae.  Figure 4 displays effects of nystatin and the negative control. In the negative control group can be observed the presence of ovoid blastospores and hyphae.

As the extract and active fraction demonstrated a similar performance, the antiproliferative test was performed only with the crude extract.* A. colubrina *demonstrated nonspecific cytostatic activity and was capable of diminishing tumor growth in all cell lines at an approximate concentration of 250 *μ*g/mL. However, the extract did not demonstrate cytocidal activity.  Table 3 displays the 50% growth inhibition values. Mean Log GI_50_ was 1.81 *μ*g/mL.

The phytochemical characterization revealed a high total polyphenol content (53.18% gallic acid equivalents). Tannin content was 8.77% catechin equivalents and flavonoid content was 0.28% quercetin equivalents.

## 4. Discussion

Despite the widespread use of* A. colubrina* by the population, few studies have sought to demonstrate its properties scientifically. The bark of this tree is reported to have effective action as an anti-inflammatory agent with peripheral antinociceptive activity, indicating the existence of compounds with potential for the development of drugs for the treatment of inflammation and pain [23]. The present findings indicate the antifungal potential of this plant, as the extract and its active fraction inhibited the growth of* Candida albicans* (MIC = 0.31 mg/mL), demonstrating strong antimicrobial activity [24].* A. colubrina* also demonstrated a fungistatic nature, with a high MFC (1 to 2 mg/mL), which suggests the inhibition of the growth of the microorganism without causing its death

*C. albicans* biofilm consists of a mixture of yeast, hyphae, and pseudohyphae [25]. In the maturation phase an increase in the growth of the hyphae and a reduction in the yeast occur [25–27]. In the SEM analysis, the* A. colubrina* demonstrated the capacity to inhibit the formation of hyphae, with a predominance of blastospores, demonstrating the susceptibility of the* C. albicans* biofilm to this plant. Infections by this microorganism have exhibited increased resistance to antifungal agents, leading to treatment failures and relapses [28]. The present findings show the possibility of developing novel antifungal agents based on* A. colubrina*, as the extract and its active fraction demonstrated the capacity to inhibit the formation of* C. albicans* biofilm, with better results than those achieved with nystatin. The cell wall plays a fundamental role in nearly all aspects of the biology and cellular pathogenicity of yeasts, acting as a permeability barrier. Its structure maintains the shape of the yeast and plays a mediating role in interactions between the microorganism and its surrounding environment [29].

After the demonstration of the antimicrobial activity of a natural product, it is important to determine whether it also has an antiproliferative effect. Drugs derived from medicinal plants have played a significant role in the treatment of cancer. Indeed, among all antitumor drugs available between 1940 and 2002, 40% originated from natural or semisynthetic products [30]. According to the criteria of the US National Cancer Institute for the classification of antiproliferative activity, a product with a mean log GI_50_ greater than 1.5 is considered inactive [31].* A. colubrina *had a mean of 1.81, indicating the absence of antiproliferative activity against the tumor lines tested. However, the extract demonstrated a tendency toward diminishing cell growth, especially leukemia-causing cells. An absence of activity was also found regarding the nontumor line (keratinocytes), indicating that the extract is not toxic to normal cells. While not demonstrating cytocidal activity, the extract was capable of inhibiting cell growth in a nonspecific fashion, which characterizes the substance as cytostatic.

*A. colubrina *also demonstrated interesting results regarding* Pseudomonas aeruginosa* (MIC = 0.5 mg/mL). The emergence of this bacterium as an opportunistic human pathogen in the last century may be a consequence of its resistance to antibiotics.* P. aeruginosa* is considered an important source of bacteremia and a predominant cause of illness and death in patients, such as those with cystic fibrosis [32].

The phytochemical analysis confirmed the abundance of phenolic compounds in* A. colubrina*, which are usually related to the pharmacological activity of plants. In the phytochemical screening of the bark of* A. colubrina, *authors have found catechins, flavonoids, phenols, saponins, steroids, tannins, triterpenes, and xanthones, suggesting the participation of secondary metabolites in the biological activity of this plant [23]. In the present study, polyphenols accounted for 53.18% of the extract. It has been suggested that these substances are capable of causing metabolic instability in* C. albicans* and destroying the enzymatic activity of proteasomes, thereby contributing to reductions in the growth rate of the microorganism as well as the formation and maturation of the biofilm [33]. The large amount of polyphenols in* A. colubrina *may explain the results found in the analysis of biofilm inhibition.

Further studies are needed to determine the mechanism of action of* A. colubrina *on* C. albicans*. The present results are promising regarding the antifungal potential of this plant. It is also important to test this substance with other species of the genus.

## 5. Conclusions

The findings of the present study suggest that* A. colubrina *has compounds with strong antifungal activity, which are capable of inhibiting* Candida albicans* cell growth and the formation of biofilm by causing considerable morphological changes in the structure of this microorganism. With regard to antiproliferative activity, the extract demonstrated the capacity to diminish the growth of tumor cells and proved inactive against normal cells. Thus, this plant has potential in the development of new drugs and its mechanisms of action merit further investigation.

## Figures and Tables

**Table 1:** Minimum inhibitory, bactericidal, and fungicidal concentrations of hydroalcoholic extract from *Anadenanthera colubrina* (Vell.) Brenan evaluated against bacterial species and *Candida albicans. *

Microorganisms	*Anadenanthera colubrina* (Vell.) Brenan	
	MIC (*µ*g/mL)	MBC/MFC (*µ*g/mL)
*Candida albicans* ATCC 18804	31.25	1000
*Streptococcus mutans* ATCC 25175	2000	>2000
*Streptococcus sanguis* ATCC 10557	2000	>2000
*Enterococcus faecalis* ATCC 29212	2000	>2000
*Pseudomonas aeruginosa* ATCC 27853	500	1000

**Table 2:** Minimum inhibitory and fungicidal concentrations of fractions of hydroalcoholic extract from *Anadenanthera colubrina* (Vell.) Brenan evaluated against *Candida albicans* (ATCC).

Fractions of hydroalcoholic extract from *Anadenanthera colubrina* (Vell.) Brenan	MIC (*µ*g/mL)	MBC/MFC (*µ*g/mL)
Ethyl acetate	31.25	2000
Hexane	62.5	2000
Chloroform	125	2000
Aqueous	1000	2000

**Table 3:** Distribution of cell growth inhibition (GI_50_) by *Anadenanthera colubrina* (Vell.) Brenan extract tested on human tumor cell cultures.

Cell lines	*Anadenanthera colubrina* (Vell.) Brenan
	GI_50_ (*µ*g/mL)
Ovary (NCI-ADR/RES)	61.3
Kidney (786-0)	142.7
Lung (NCI-H460)	80.3
Ovary (OVCAR-3)	131.1
Colon (HT29)	95.8
Leukemia (K562)	5.7
Keratinocytes (HaCaT)	99.7
Mean log GI_50_	1.81
